# The effect of AI on pink marketing: the case of women’s purchasing behavior using mobile applications

**DOI:** 10.3389/frai.2024.1502580

**Published:** 2024-11-18

**Authors:** Hasan Beyari

**Affiliations:** Department of Administrative and Financial Sciences, Applied College, Umm Al-Qura University, Mecca, Saudi Arabia

**Keywords:** artificial intelligence, women’s purchasing behavior, mobile applications, consumer engagement, Saudi Arabia, pink marketing

## Abstract

This research looks in detail at the dynamics of pink marketing and its effect on the purchase behavior of Saudi women through mobile applications, with an emphasis on Artificial Intelligence (AI) as a moderator. Furthermore, this study assesses the effects of customized pink marketing strategies – product, price, promotion, and place – on buying intentions and behaviors. A closed-ended questionnaire was adopted to measure constructs associated with women’s mobile app purchase behavior influenced by pink marketing and AI elements. Structural Equation Modeling (SEM) was the study tool used to examine how AI affects women’s consumer behavior and how it influences pink marketing. The results suggest that each component of the pink marketing mix significantly influences buying behavior, especially price and promotion. Additionally, AI has a significant moderating effect, improving the personalization and effectiveness of marketing activities. The results of this study highlight the essential role of AI in forming consumer engagement in the digital market, providing useful input for marketers who intend to target women in Saudi Arabia. This study complements the understanding of gender marketing in the digital era and provides a vision for the possibility of AI fundamentally changing traditional approaches.

## 1 Introduction

The rapid growth of the e-commerce sector in Saudi Arabia has resulted in fundamental changes to women’s shopping habits. Reportedly, this has resulted in 60% increase in online shopping between 2018 and 2023 (Al Hamli and Sobaih, 2023). While e-commerce is a small part of total retail sales, this can only mean that there is still plenty of room for its growth to expand. Marketing strategies that are pink and focus exclusively on women have become even more sophisticated and complex, adapting to the multiplicity of demographics and interests of women in Saudi Arabia. One of the main factors that has led to the redefinition of women’s consumer engagement is AI inclusion in pink marketing. For example, mobile commerce among Saudi women has increased by 56% within the past 12 months (Alawadh and Barnawi, 2024). This portrays the effect of AI on women ‘s buying behavior and its application in mobile applications.

The main objective of this study were the research questions which were designed to probe the sizes of the components affected by AI: price, product, promotion, and place. The objectives were twofold: to measure the impact of AI on the effectiveness of pink marketing and to see how Saudi women’s relationship with AI-driven shopping platforms is in terms of nuance. The value of this study lies in its attention to emerging markets. Many studies have focused on developing markets; therefore, they often miss the specific cultural and technological environments of a country such as Saudi Arabia. This research fills this gap by offering concrete data on the role of AI in a flourishing industry whose models are rapidly changing, especially among female consumers.

The reason for the selection of four dimensions consists of price, product, promotion and place, because of its conventional reality of marketing mix influences customers (Shehata and Fayyad, 2020; Zarei and Kharajo, 2023). The 4Ps model of marketing has been a long-standing theoretical foundation for marketers, providing an organized framework for exploring how different marketing elements interplay to shape consumer choices. This study examines the impact smartphone application of each of these dimensions on the purchase behavior of Saudi women within the context of pink marketing. The 4Ps framework has been widely used in previous research to explain general consumer behavior, but limited research has looked at how these factors influence the purchasing behavior of women in a culturally specific context, in our case Saudi Arabia. In this study, we contribute to filling this gap by applying the 4Ps to this segment of the population who are often overlooked in the digital commerce literature and offer insights into how they respond to targeted marketing strategies.

Additionally, this attention addresses shortcomings within previous research on pink marketing, which tend to focus on broader strategies, without consideration to the historical and economic realities of emerging markets. Although there are many studies that have identified the relevance of targeted marketing strategies aimed at female shoppers, most fail to reflect on appropriate price-product-promotion-distribution channels in relation to the buying behavior of that market body in the Middle Eastern context. This study expands part of the traditional 4Ps model in the context of the pink market by analyzing these dimensions in Saudi Arabia, as well as demonstrates how cultural drivers and the acceptance of technology converge in the Saudi pink market in relation to pink marketing with special emphasis on mobile shopping perspective. By doing so, it allows a clearer perspective as to how these aspects motivate this demographic when it comes to consumer engagement and purchase intentions.

In doing so, this study goes beyond the limitations of the existing literature, which has on several occasions overlooked the subtlety of developing countries, particularly those in the Middle East. The key contribution of this article is the connection between modern marketing theory and practical AI applications within the relevant cultural context of Saudi Arabia. Consequently, the latter is not solely innovative, but also vital in comprehending how AI can increase the performance of women-targeted marketing campaigns. This part of the paper will be set out in a unified, argumentative manner, starting with a theoretical foundation, which will be followed by an empirical analysis. The systematic approach is described, followed by the examination and analysis of the results. Consequently, the implications are understood, limitations are recognized, and future directions for research are put forward. Each section is cumulative, and in the end, whole-body work is supposed to demonstrate a better understanding of AI’s role in pink marketing development in the Saudi market.

## 2 Literature review

The concept typically refers to the female-oriented development of products and services aligned with women’s unique preferences and buying behavior. In a broad sense, pink marketing is defined as more than just a business that uses pink, but rather as a methodology that addresses the emotional, psychological, and practical aspects of women’s consumption needs (Massoudi, 2020; Hussien et al., 2024). The study above argues that the technique can be successful, but it has to be utilized with care lest stereotypes might lead to the disillusionment of consumers. Zidan (2020) suggested that the notion of pink marketing should be multifaceted, which includes providing valuable messages in the context of female roles and the important issues they face in society. Moreover, the authenticity of the pink campaign was also mentioned in some research materials; thus, the campaign was to be backed up by a genuine goal for women’s problems and interests (Shehata and Fayyad, 2020; Zarei and Kharajo, 2023). As Hussien et al. (2024) pointed out, the use of pink marketing should be more profound than on the surface, whereby women are not simply treated as a uniform physiological group but as a group with multiple kinds of complexities. This platform of knowledge lays the foundation for learning about pink marketing, and it can undoubtedly be powerful if used carefully and with a sense of depth.

The marketing mix, comprising product, price, promotion, and place, is the dominant and classical approach that has been the basis for formulating market strategies. This serves as a framework for companies to make their market offer to the public (Elgarhy and Mohamed, 2023; Warrink, 2015). This framework is a model developed through past time tests. It has been quite a valuable instrument for marketers so far, showing where to put certain products and how to sell them. To this day, the 4Ps – product, price, promotion, and distribution – are referred to by both scholars and practitioners as a core concept that informs product design, as it needs to meet consumer needs and wants and is priced to reflect its value and competitiveness. On the other hand, it markets the product to raise consumer awareness, stimulate demand, and make consumers aware of where to buy the product. The model has also been criticized and developed with the emergence of the digital age; however, it remains the basis for understanding the complicated mechanism of market dynamics.

The product part of the marketing mix is made more granular than what usually exists when pink marketing is integrated into it because it focuses on developing and promoting goods that are tailored for the female segment to which they are marketed. Pink marketing implies the ability to deeply understand the minds, preferences, and values of female consumers, which should be followed by producing products that match their psychological and cultural backgrounds (Vitsentzatou et al., 2022). Along with the authors, (Budianto et al., 2023; Rosário and Raimundo, 2021) pointed out that in celebrating female delineation, functional and youthful marketing is drawn to women of correct product development. It addresses the practical needs of the target audience along with their aspirations. To this extent, other researchers have established that any product marketed to women must signify the brand’s broad pursuit of social values that relate to the target audience, in addition to the sustainability and ethics of production, which are needed for a more meaningful, long-term relationship with the audience (Niazi et al., 2021). Hence, product strategy, like pink marketing, does not have a product-specific strategic scope. Instead, it is an integrated approach to position a specific product as appropriate to the complex system of requirements, expectations, and social roles for a woman.

With respect to pink marketing, pricing policy is an essential factor, suggesting not only the economic value of the product but also the meaning and importance that the audience takes into account while purchasing. At the same time, price is an essential factor in decision-making by female consumers, with premium pricing used by the brand to reflect the high quality and exclusivity perceived by them (Sukanta and Muis, 2022). In addition, this part of pricing should be handled carefully because it can change consumers’ conceptions of trademarks and their personal identity (Kung et al., 2021). According to various studies, women are known to be fervent of paying a higher amount for products exclusive to their packaging attributes and values, such as ethical sourcing and genuineness (Niazi et al., 2021). In addition, the terms and conditions of pricing are transparent, and offering low prices and value for money can generate trust and loyalty among female consumers (Kung et al., 2021). That is why the price in pink marketing is not only a matter of cost, but also an invaluable connection between a brand and its female customers. As such, the brand establishes an emotional bond with its female customers.

In ‘pink marketing,’ the idea of ‘place’ expands as the female-dominated, both physical and digital areas are created where products are sold. The strategic placement of products that often overlaps with women’s desire for convenience and comfort by emphasizing accessibility and security draws more female consumers (Elgarhy and Mohamed, 2023). Using this approach, we find products offered in locations that women feel comfortable and often visit, both in physical stores and online platforms that focus on the shopping traits of women (Budianto et al., 2023). As the virtual space is changing, ‘place’ continues to refer to the broader digital area, which covers e-commerce, mobile shopping and social media, where women can get close to brands (Niazi et al., 2021). Evidence of the pink marketing framework given by the importance of ‘place’ expresses the importance of brands being in the preferred context and time, taking on the lifestyle and preferences of women for a smooth shopping process.

Pink marketing promotion is a strategic plan that covers more than just advertising. Instead, it is a communication channel between the brand and its female target market. It involves information that touches the emotional and cultural needs of its audience in a more profound and personal way (Elgarhy and Mohamed, 2023). Pink advertising is therefore about storytelling and developing narratives that agree with the feelings, expectations, and values of women. According to Sukanta and Muis (2022), the promotional strategy applied in this situation should take the form of campaigns that embrace and celebrate diversity, offering women stories to which they can attach themselves. Vitsentzatou et al. (2022) advise a conversational and engagement-driven approach, and are trying this out through popular social media and engaging influencers to foster two-way communication with consumers. The objective is to move to a different level, where instead of meekly pushing products with regular promotions, businesses engage in a brand experience that brings out the unique paths of women as opposed to the paths we are made to take by vendors. Consequently, in pink marketing, promotional activities are accentuated to inform, persuade, and arrange women’s customer base.

The penetration of artificial intelligence (AI) into pink marketing channels is a groundbreaking factor in marketing as it introduces new methods of audience interaction. AI technologies assist in gaining better knowledge of consumer tendencies and likes/dislikes, which makes it easier to design marketing strategies that have a personal touch and match/align better with the female audience (Nawaz et al., 2021). AI can refine customer segmentation procedures by computing massive datasets and uncovering different consumer clusters more accurately for women. They can thereby fashion product lines and their messages to meet the particular wants and needs of various groups of women and provide a better focus on marketing efforts through additional efficiency (Beyari and Garamoun, 2022; Cha et al., 2019). AI will be capable of analyzing the customer journey, spotting the main touchpoints that impact buying decisions, and assessing client behavior, thus helping companies adjust their marketing strategies accordingly and better remain coherent with their female clients’ expectations and lifestyles. In addition, AI facilitates the personalization of marketing communication channels, enabling the analysis of past customer behavior and the provision of individual customer content and recommendations, thus improving customer engagement and bringing brands and consumers closer together (Ziakis and Vlachopoulou, 2023). The technology uses AI tools, such as chatbots and virtual assistants, as a part of communication to present customers with instant interactive conversation options that are fully customized for the female target group, thereby improving service and support for female users of the product.

The purchase activity of women on smartphones as a subject of interest for marketers has become a central point because sales of mobile commerce have been very responsive, particularly among female consumers. Researchers found that women are more likely to use mobile phones for shopping, which is attributed to convenience as most women can shop on the go, whereas the ability to shop while on the go is not associated with men’s shopping browsing (Tengli and Srinivasan, 2022). Moreover, mobile commerce has become especially attractive to female shoppers because it provides a more personalized touch, including individual advertising, offers, and promotions, which are usually tailored to reflect purchasing history and preferences (Borres et al., 2023). With mobile platforms integrated, the social aspect of purchasing, such as showing potential items for feedback to friends and family, becomes a more engaging and interactive affair (Carfora et al., 2021). On the contrary, mobility’s security features such as biometric authentication, such as the fingerprint scan, are one of the measures that have developed trust among female consumers who are very cautious when it comes to online transactions (Amirtha et al., 2020). Hence, mobile devices offer women a unique opportunity to save time, individualize their purchases, create a social bond, and share their purchases with others, which makes mobile platforms the most common among women when it comes to product exploration and purchase.

## 3 Theoretical background

### 3.1 Theory of planned behavior

Appropriating the Theory of Planned Behavior (TPB) in the study’s framework will ensure a solid psychological basis for understanding all factors negatively affecting Saudi women’s mobile app purchasing. The TPB hypothesis states that behavioral intentions that shape attitudes, subjective norms, and perceived behavioral control predict the likelihood of a person engaging in a specific behavior (Ajzen, 1991). In addition, this theory may be applied by showing that attitudes towards pricing, products, promotions, and places affect women’s intentions to purchase via mobile applications. Besides the cognitive factors of norms, the subjective factor may have a plentiful influence, especially if we realize social media’s effect on the target group, which has been proven in the model as well. This leads to the last factor, perceived behavioral control, which indicates whether a woman finds it easy or difficult to make purchases using mobile applications. The moderating effect of this factor can be linked to AI-aided features such as personal recommendations and virtual assistance, suggesting a complex interplay between technology and consumer psychology. Thus, the TPB perspective of how pink marketing components, AI, and sales behavior influence each other can be examined and grasped as a result of this analysis.

### 3.2 Theory of reasoned action

**Figure 1:**
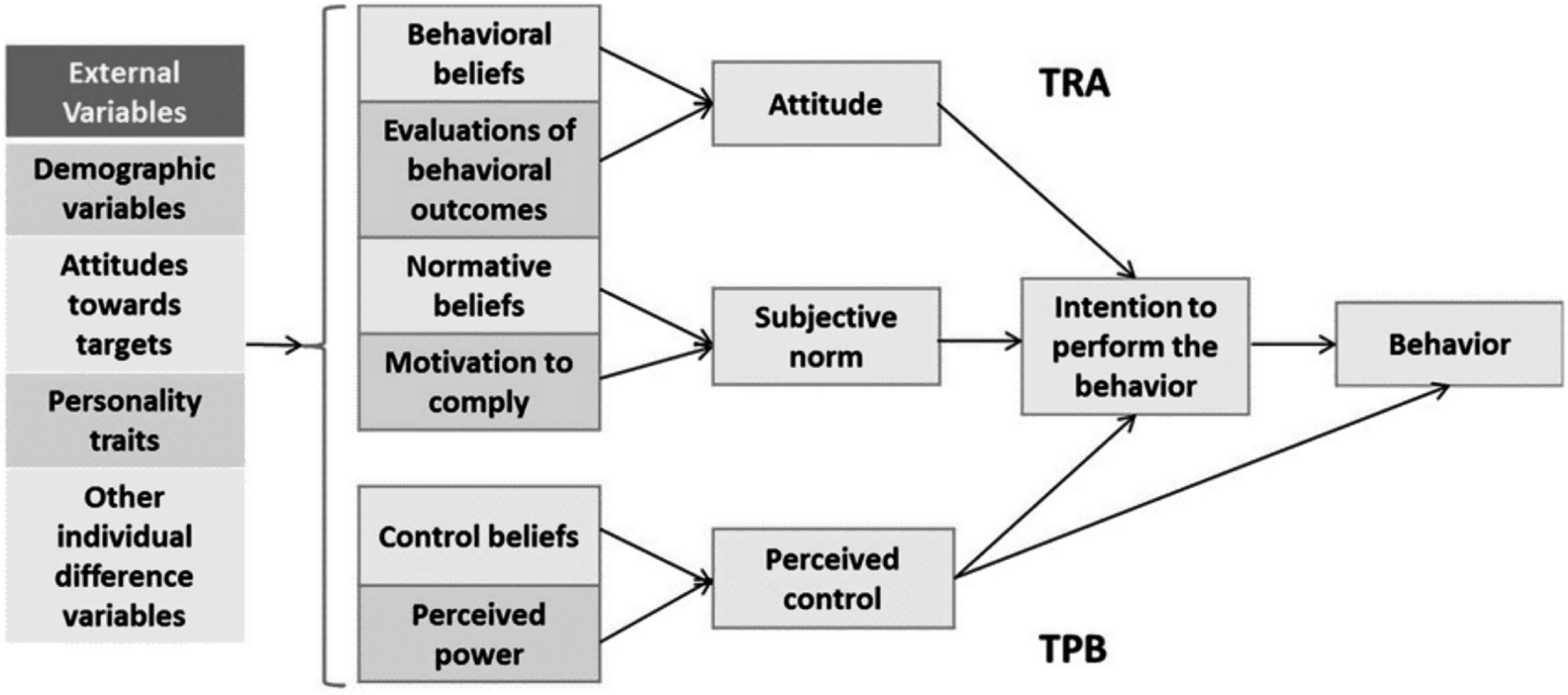
Combined theories of reasoned action and planned behavior (Al-Suqri and Al-Kharusi, 2015).

The Theory of Reasoned Action (TRA) postulates that an individual’s behavior is determined by their intention to perform the behavior, as shown in Figure 1. Behavior is also influenced by attitudes towards behavior and subjective norms (Ajzen and Fishbein, 2007). Within the context of this study on Saudi women’s mobile application purchasing behavior, the TRA provides a theoretical basis for understanding how women’s attitudes towards pink marketing (encompassing price, product, promotion, and place) and subjective norms (such as cultural expectations and the influence of social networks) shape their intentions and, consequently, their actual purchasing decisions. The AI components in the conceptual model—personalized recommendations, targeted advertising, chatbots and virtual assistants, and social media influence—can be seen as modern extensions of these subjective norms and attitudes, where AI shapes the informational and normative beliefs that underlie purchasing behavior. Thus, the TRA offers a vital perspective for analyzing the causality that leads from pink marketing and AI influences purchase intentions and behaviors (Al-Suqri and Al-Kharusi, 2015). This theoretical backdrop enriches the study by highlighting the cognitive underpinnings of the decision-making process of Saudi women engaging in mobile commerce.

### 3.3 Research model

**Figure 2:**
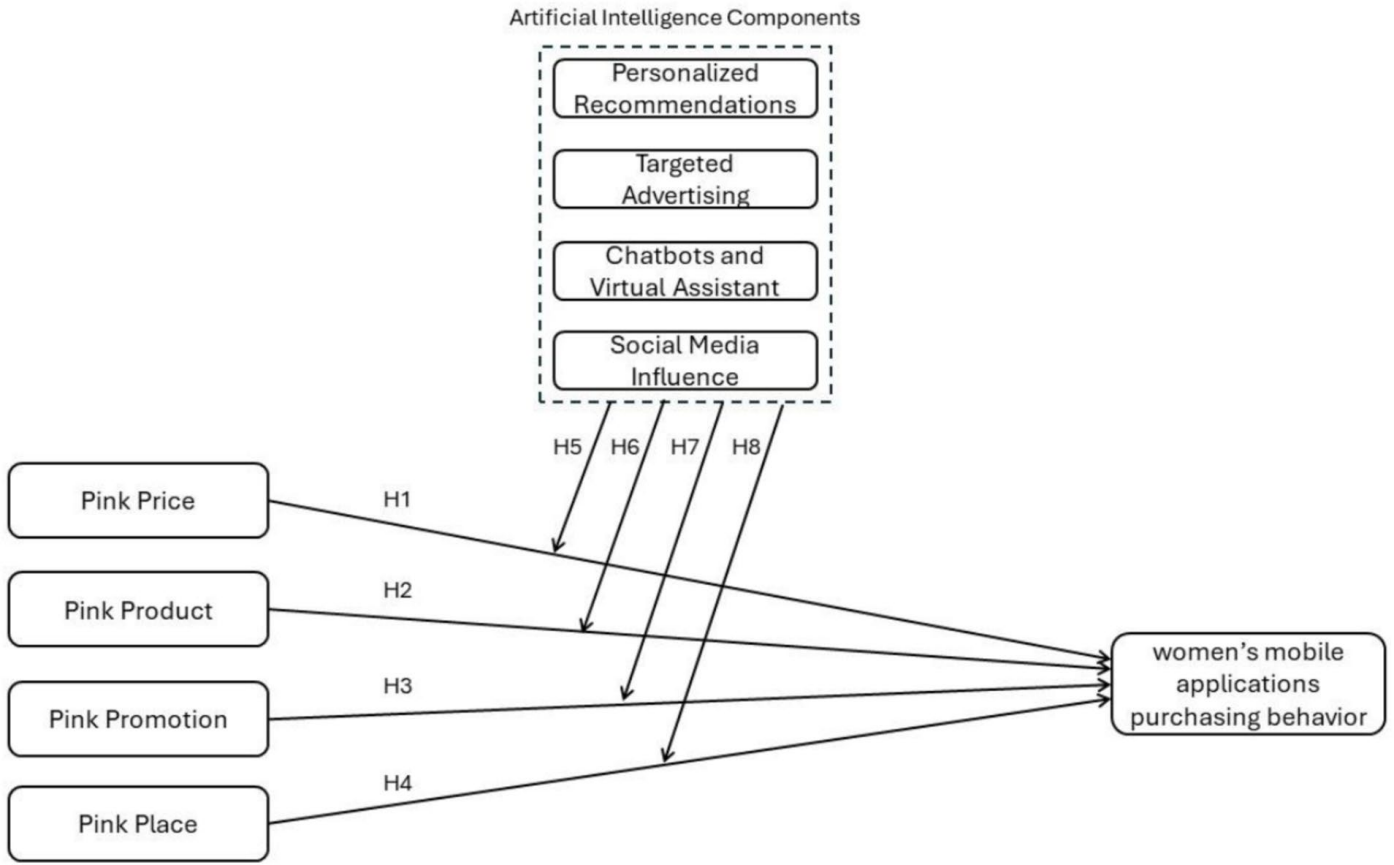
Conceptual framework.

Within our research framework, the pink marketing model has significant components, including price, product, promotion, and place, that affect how women in Saudi Arabia purchase through mobile apps (Figure 2). It blends the moderation capabilities of AI using features such as personalized recommendations, targeted advertisements, chatbots, virtual assistants, and social media effects, which are among the key factors in modern IOT. Crunching these theories through the Theory of Planned Behavior and the Theory of Reasoned Action compares the link between their beliefs, accepted behavioral control, and societal norms to their consumption behavior and consciousness. AI-specified components are supposed to reinforce or transform these interrelationships through the idea that technology can either strengthen or change the traditional relations of the marketing mix on consumer behavior.

## 4 Hypothesis development

The assumption that the color of the pricing influences one’s choice to buy a women’s mobile app is based on the idea that women are more sensitive to value and price while making purchase decisions. Previous research has shown that price influences consumer choice, although the gender-specific marketing strategy approach also contributes (Borres et al., 2023). Female consumers have shown a separate price-value judgment, followed by weighing the costs, quality, and emotional aspects involved (Amirtha et al., 2020). In mobile shopping, the convenience of price comparison and instant choices could be a function that makes marketers tread pink pricing strategies carefully (Carfora et al., 2021). Thus, we need to understand the link between pricing and consumer psychology. This implies that we should study how pink pricing, which reflects the strategy aimed at meeting women’s preferences and the amount they are willing to pay, affects buying on mobile shopping sites. Thus, this study aimed to empirically investigate this relationship, giving rise to the following hypothesis:

*H1: Pink price has an impact on women’s mobile applications purchasing behavior.*

The role of pink products in the mobile application engagements of female shoppers is being investigated, particularly connecting a product’s design and marketing process to the group of buyers. This concept stands for the fact that some goods are produced by considering the characteristics of consumers of a specific gender. Design, function, and brand can be considered in this context (Sohaib et al., 2018). Research findings indicate that female consumers who see themselves as reflected in products and who view their values within the products are highly likely to buy them (Andijani and Kang, 2022). In addition, the product relevance and resonance issue becomes more important when talking about mobile applications, since digital engagement can be more personalized and immediate (Amirtha et al., 2020). The direct link between pink products and mobile shopping is perceived to be robust because mobile applications can provide personalized product suggestions or targeted marketing. Therefore, based on insights from the literature, the following hypothesis is proposed.

*H2: Pink products have an impact on women’s mobile applications purchasing behavior.*

With the saturation of pink marketing promotion in the digital environment, the direct impact on women’s mobile app buying behavior can be seen as a marquis-type marketing strategy meant to target the female audience. Among the pink marketing types, promotion is a defined category of intelligent strategies for talking to and persuading female consumers about the available products and services in a manner that caters to females. However, mobile platforms are the most influential, where marketers advertise in a more targeted form that takes advantage of user data to improve relevance and appeal (Andrlić et al., 2023). Moreover, studies have shown that marketing platforms that strike a chord and accommodate and represent women’s experiences and concerns are most likely to influence buying behavior (Evangelista et al., 2022). The effects of such promotions are exaggerated because mobile devices are all around them, and real-time interactions with the audience occur. Hence, it can be said that there exists a strong relationship between promotions and purchasing behavior. Therefore, considering the strategic importance of promotional activities in driving consumer behavior, especially within the context of personalized mobile experiences, we hypothesize the following.

*H3: Pink promotion has an impact on women’s mobile applications purchasing behavior.*

The term ‘place’ has stretched its physical definition over and beyond the digital zone within which the transactions take part. The main pros, including the convenience, accessibility, and context of mobile platforms, are thought to be in action in this space (Lerro et al., 2019). The ‘place’ created by the digital environment will have a significant impact on women’s choices as mobile apps have been successfully integrated into their day-to-day activities, and the personal nature of interactions in apps sets mobile apps favorably in comparison to traditional and online shopping environments (Kim and Oh, 2020). Research points to the fact that the perception of accessibility and availability largely dictates the likelihood of a particular product reaching out to consumers, and that this applies mainly to women who pay particular attention to the fact of whether buying this or that is supposed to be safe, time-saving, and quick (Elgarhy and Mohamed, 2023). As the evolving definition of ‘place’ in digital commerce and its influence on consumer purchasing behavior are considered, we hypothesize the following:

*H4: Pink place has an impact on women’s mobile applications purchasing behavior.*

The moderating effect of artificial intelligence (AI) on the relationship between pink prices and women’s buying behavior in mobile applications is a significant problem facing the current research space. Using AI technologies, marketing strategies can be adjusted in real time and even offer coupons and discounts customized for individual consumer needs and behavior. These technical pricing mechanisms, which exploit AI, can progressively make goods and services more enticing to women when such products are priced in line with women’s individual price sensitivity and perceived value (Nawaz et al., 2021). Moreover, AI can process considerable datasets to find the best prices and those that maximize conversion rates among female consumers on separate mobile platforms (Lashin et al., 2022). Through the role of AI in moderating the pink pricing process, buying behavior can be either heightened or tranquilized, depending on the approach, which influences the target audience’s perception (Qin et al., 2022). AI’s forecasting and machine learning will fine-tune pricing strategies and help match consumer expectations, implying AI’s complexity and causality in this relationship. Thus, we hypothesize as follows:

*H5: AI has a moderating effect on the relationship between pink price and women’s mobile applications purchasing behavior.*

AI plays an especially conspicuous role in the connection between the likability of a product and a customer buying it in pink marketing. Based on data collection, AI technologies can allow customized product selection and enhance product relevance for women to have a mobile application. AI has penetrated virtual trial rooms, augmented reality, and uniquely personalized product recommendations. These features can broadly transform how women who use products perceive and relate themselves to them (Jo, 2022). Additionally, the data accumulated by AI analysis can ensure that creator input and feedback will be reviewed faster than before, and theology will be amended to meet changing consumer needs (Bueno and Gallego, 2021). The utilization of AI in a delicate manner provides the ability for products to correspond better with the values and demands of women, which leads to the idea that AI plays a striking role in the causal relationship between pink products and buying behavior. Hence, considering the transformative capabilities of AI in product marketing and its alignment with consumer preferences, the following hypothesis is proposed:

*H6: AI has a moderating effect on the relationship between pink product and women’s mobile applications purchasing behavior.*

AI has the potential to make it far easier for firms to promote products among women on mobile apps because of its impact on women’s future buying habits. Artificial intelligence helps deliver personalized marketing communications, match promos with tariffs in the media timetable, and optimizes ad content to an individual’s preferences, which, as a result, can increase the effectiveness of the entire advertising campaign (Cabrera-Sánchez et al., 2020). As a case in point, a machine learning algorithm considers purchasing histories and online behaviors for different segments of the women’s audience. It uses these to determine the most efficient marketing technique for each, thus increasing the likelihood of buying. In addition, AI-enabled tools may connect women and consumers through interactive and personalized content. Moreover, the possibility of an AI that can make decisions regarding promotions on the fly by observing consumer behavior points to AI’s moderating role in strengthening the roles of the pink promotion approach (Chen et al., 2020). Given the advanced capabilities of AI to enhance the personalization and responsiveness of promotional activities, it is hypothesized that.

*H7: AI has a moderating effect on the relationship between pink promotion and women’s mobile applications purchasing behavior.*

Artificial intelligence (AI) provides a critical moderation factor between pink places and women’s purchase behavior in mobile applications. AI technologies improve the ‘digital space’ by maximizing user interface efficiency and personalizing the user experience. Thus, women feel that online shopping environments are more intuitive and responsive to their preferences (Nawaz et al., 2021). For example, AI can adapt the mobile app layout to real-time browsing and purchasing history to ensure that it is served with products it is likely interested in Massoudi (2020). Furthermore, AI provides VR and AR features whereby women can see the products through VR glasses or their smartphones and try them out as they would be an actual product, increasing their chances of purchasing (Al Hamli and Sobaih, 2023). This tendency of AI to amalgamate the idea of place drastically changes how products are supplied and experienced, and thus influences purchase decisions. Given these enhancements, the following hypothesis is proposed.

*H8: AI has a moderating effect on the relationship between pink place and women’s mobile applications purchasing behavior.*

## 5 Methodology

### 5.1 Item measurement

We used an organized method to measure different constructs concerning women’s purchase behavior on mobile applications, influenced by pink marketing and AI elements. We used a closed-ended questionnaire consisting of 24 questions distributed across six constructs, as shown in Appendix A. These questions were aimed at evaluating particular dimensions of consumer behavior and attitudes and were scored on a Likert scale ranging from 1 (strongly disagree) to 5 (strongly agree). This type of scale format provided us with the possibility to measure in numbers which high-level viewers would agree or disagree with the offered statements and respond to them on a systematic basis. Questions allocation was different among the constructs: the minimum number of questions per construct was three and the maximum was five.

### 5.2 Population, sampling, and data collection

The study targeted a specific demographic group, which is necessary to guarantee the relevance and viability of the findings. The demographic profile was limited to Saudi women aged 18 and over who were active mobile device users and had a record of getting the items through the online purchase apps. The >18-years age group was targeted because it gives unbiased opinions about AI-operated brand marketing that is pink-inspired. The field study focused on frequent shoppers or those who use mobile apps to ensure the selection of users with the same interest as the studied phenomenon. More specifically, given the above demographic selection, the expected number was estimated to be approximately 4.96 million (GASTAT, 2023). In addition, the geographical location was one of the top critical points that were considered, which comprised urban areas, especially Riyadh (Middle), Makkah (Western), and As-Sarqiyah (Eastern). Participants’ socioeconomic status was considered, as only some from different socioeconomic positions may respond similarly to pink marketing tools and AI integration. Ultimately, the research was conducted with a sample size of 309 participants who offered divergent opinions on the questionnaire items. Moreover, the deanship of scientific research committee in Umm Al-Qura University ethically approved the research with its inform consent. Additionally, the study has obtained a written informed consent for participation.

### 5.3 Research approach

We employed a Structural Equation Modeling (SEM) approach to explore the effect of artificial intelligence on pink marketing and its impact on the purchase behavior of Saudi women concerning mobile applications. SEM’s strengths include its ability to analyze intricate relationships between observed and latent variables and its use in consumer behavior, marketing effectiveness, and technology interaction (Kline, 2023). While attitudes and preferences are critical to understanding consumer behavior in digital environments, this method is particularly good at evaluating latent constructs. The ability of SEM to handle many dependent relationships simultaneously allows us to analyze the potential moderating roles of AI in pink marketing elements, thus identifying both direct and indirect effects on purchase behavior. We conducted the analysis using IBM SPSS AMOS because it provides advanced functionalities in structural equation modeling. IBM SPSS and Microsoft Excel were used for general statistical analyses and data manipulation. Such techniques create a thorough analysis, delivering reliable results for strategic decision-making and policy formulation in digital marketing towards women in Saudi Arabia.

## 6 Data analysis

### 6.1 Demographic analysis

The age profile of the female respondents to the study indicated a dominance in the 31–40 years category, accounting for close to half of the participants at 47.9% (Table 1). The 18–30 years of age group accounts for 23.9%, and the section >40 represents 28.2% of the respondents, meaning that middle-aged individuals are the majority. The demographic distribution indicates that a considerable part of mobile application buying behavior can be attributed to those in the middle of their careers and probably at a steadier financial life stage. Such understandings offer a unique perspective on age-related subtleties in buying behavior, and by their means, pink marketing strategies that are designed to target age-specific consumer patterns can be customized.

**Table 1:** Distribution of respondents by age.

Category	Frequency	Percent
18–30 years	74	23.9
31–40 years	148	47.9
>40 years	87	28.2
Total	309	100.0

Respondents with different educational levels created a diverse situations, with a significant share of high school diplomas (40.5%). Table 2 shows that 28.2% of the surveyed people had a college education and 22% had a university level education, proving that most female participants were educated up to at least the secondary stage. The segment without a formal education was the smallest. The diversity of the number of children exposed to and aware of these platforms on varying degrees is to be expected with the different levels of influence the AI-supported shopping experiences would have.

**Table 2:** Distribution of respondents by education level.

Category	Frequency	Percent
Uneducated	29	9.4
High School	125	40.5
College	87	28.2
University	68	22.0
Total	309	100.0

As for the geographical distribution, the respondents were mainly from Makkah at 45%, with Riyadh coming next at 39.2%. The As-Sarqiyah region contributed to the percentage of 15.9%. This distribution helps capture whether regional-specific factors, such as local culture, urbanization, and economic situation, influence pink marketing and the trend of mobile application purchases. Because of the closely located number of participants in the cities, this analysis demonstrated a high level of mobile commerce engagement that depicted the essence of mobile marketing within pink marketing. Additionally, the presence of all participants in these locations underscores the application of location-specific pink marketing strategies (Table 3).

**Table 3:** Pink marketing distribution of respondents by different cities.

Category	Frequency	Percent
Makkah	121	39.2
Riyadh	139	45.0
As-Sarqiyah	49	15.9
Total	309	100.0

### 6.2 Assessment of the measurement model

#### 6.2.1 Indicator/composite reliability

It can be clearly seen that the factor loadings and squared loadings were significant. The loadings for Pink Product, Pink Price, Pink Promotion, Pink Place, Artificial Intelligence, and Women Mobile Application Purchase Behavior constructs ranged from substantial to high, revealing a strong relationship between each of the items and the intended constructs. The pink place factor is characterized by an outstanding indicator reliability score and an apparently good fit of the items. This demonstrates its constructs’ high face validity and outstanding representativeness. Here, the average variance extracted (AVE) is well above the threshold of 0.7 (Hair et al., 2021; for each construct separately), indicating that each construct accounts for most of the item variance. The error variance was insignificant in terms of accuracy; thus, it was within acceptable limits. Table 4 demonstrates that the aggregated average of the squared loadings amounts to 0.883, exceeding the required value of 0.7, suggesting high internal consistency. This means that the questions achieved their intended purpose.

**Table 4:** Research constructs indicators.

Construct	Items codes	Factor loading	L^2^	Error variance	Composite reliability	Indicators	Cronbach’s alpha	AVE
Pink product (PPRD)	PRD1	0.887	0.786769	0.213231	0.923	0.958	4	0.63
	PRD2	0.841	0.707281	0.292719				
	PRD3	0.921	0.848241	0.151759				
	PRD4	0.812	0.659344	0.340656				
Pink price (PPRC)	PRC1	0.983	0.966289	0.033711	0.883	0.857	3	0.73
	PRC2	0.752	0.565504	0.434496				
	PRC3	0.788	0.620944	0.379056				
Pink promotion (PPRO)	PRO1	0.901	0.811801	0.188199	0.843	0.888	3	0.62
	PRO2	0.761	0.579121	0.420879				
	PRO3	0.733	0.537289	0.462711				
Pink place (PPLC)	PLC1	0.858	0.736164	0.263836	0.897	0.964	5	0.64
	PLC2	0.803	0.644809	0.355191				
	PLC3	0.899	0.808201	0.191799				
	PLC4	0.780	0.6084	0.3916				
	PLC5	0.629	0.395641	0.604359				
Artificial intelligence (AI)	AI1	0.738	0.544644	0.455356	0.864	0.931	4	0.75
	AI2	0.926	0.857476	0.142524				
	AI3	0.632	0.399424	0.600576				
	AI4	0.820	0.6724	0.3276				
Women mobile applications purchase behavior (WPB)	WPB1	0.794	0.630436	0.369564	0.888	0.959	5	0.61
	WPB2	0.712	0.506944	0.493056				
	WPB3	0.789	0.622521	0.377479				
	WPB4	0.853	0.727609	0.272391				
	WPB5	0.760	0.5776	0.4224				

#### 6.2.2 Internal consistency

There was substantial homogeneity of the constructs, as confirmed by this study’s high Cronbach’s alpha. An alpha value of ≥0.7 or higher is acceptable, as per the observation of Rosseel (2012). The Pink Product and the Pink Place constructs had almost perfect accordance, as revealed by their alphas, which were equal to 0.958 and 0.964, respectively, as shown in Table 4. The Women Mobile Applications Purchase Behavior Construct indicated outstanding internal consistency (Cronbach’s alpha = 0.959). The Pink Price and Promotion presented in the study were reinforced with an alpha of 857 and 888, respectively. The artificial intelligence factor showed a high alpha coefficient of 0.931, which measures a high internal specification level. The summed Cronbach’s alpha for the model was 926. This statistic is a significant value that indicates high reliability of the measurement instrument. The total number of observed items was 24, which shows the confidence of the constructs, and the scale was free from false claims. The inner strength of each construct within this model reflects well on the studies on validity, and the studies performed will have sound results.

#### 6.2.3 Convergent validity

Table 4 shows the convergent validity for each construct using the Average Variance Extracted (AVE) statistic. Convergent validity has a cutoff mark at the 0.6 mark (Kline, 2023). The AVE is calculated as the sum of the squared loadings of the indicators, and the result is divided by the number of indicators. The AVE for the pink product is equal to 0.63 for ‘pink product,’ implying that it is just over the threshold, and the majority of the variance in the indicators can be explained by the central construct, that is, the concept of the pink product. All the other independent variables achieved values that were more significant than the required threshold. We also note that the AI factor has an Average Variance Extraction (AVE) above the threshold, which equals 0.75. The AVE for women’s purchase behavior on mobile applications was slightly above the required AVE of 0.61. These findings provide confidence in the measurement model’s ability to capture the constructs of interest accurately.

#### 6.2.4 Discriminant validity

Discriminant validity is obtained by displaying that the square root of each construct’s Average Variance Extracted (AVE). For this to be determined, it must be greater than the correlations between that construct and the rest of the constructs (Pearl, 2012). It seems that the main diagonal, intended to hold the square root of the AVE for every construct (pink price–PPRC, pink product–PPRD, pink promotion–PPRO, pink place–PPLC, Artificial Intelligence-AI and Women Mobile Applications Purchase Behavior-WPB), is larger than the off-diagonal passes to the rows and columns. The Table 5 shows that PPRD’s value of 0.763 is the highest among all its correlations (ranging from 0.374 to 0.617), as are other constructs, where AI has a value of 0.818 and WPB is 0.826. This pattern reveals that the variances of these measures are shared more with each other than with the measures of other constructs. Therefore, the discriminant validity for all variables was affirmed. The observed values demonstrate that the constructs are unique and differ from one another. Consequently, the measurement model was validated along with the reliability of the constructs, which were ensured as separate units within the study.

**Table 5:** Results of discriminant validity.

	PPRD	PPRC	PPRO	PPLC	AI	WPB
PPRD	0.763					
PPRC	0.617	0.699				
PPRO	0.495	0.599	0.888			
PPLC	0.608	0.482	0.322	0.722		
AI	0.555	0.386	0.328	0.436	0.818	
WMPB	0.374	0.556	0.627	0.481	0.606	0.826

### 6.3 Effect of pink product, price, promotion, and place

Different aspects of pink marketing are studied in the given path diagram to determine the factors affecting women’s purchasing behavior (WPB) on mobile applications. The path coefficient from the Pink Product (PPRD) to WPB is 0.12, indicating a positive but weak relationship. This suggests that contributory product-related factors targeted at women may not be the most potent predictors of buying behavior among mobile applications. The path coefficient from PPRC to WPB was 0.51, indicating a moderate-to-strong positive effect. This is the most powerful predictor among the four pink marketing components, which means that pricing strategies significantly affect women’s purchasing behavior on mobile applications.

**Figure 3:**
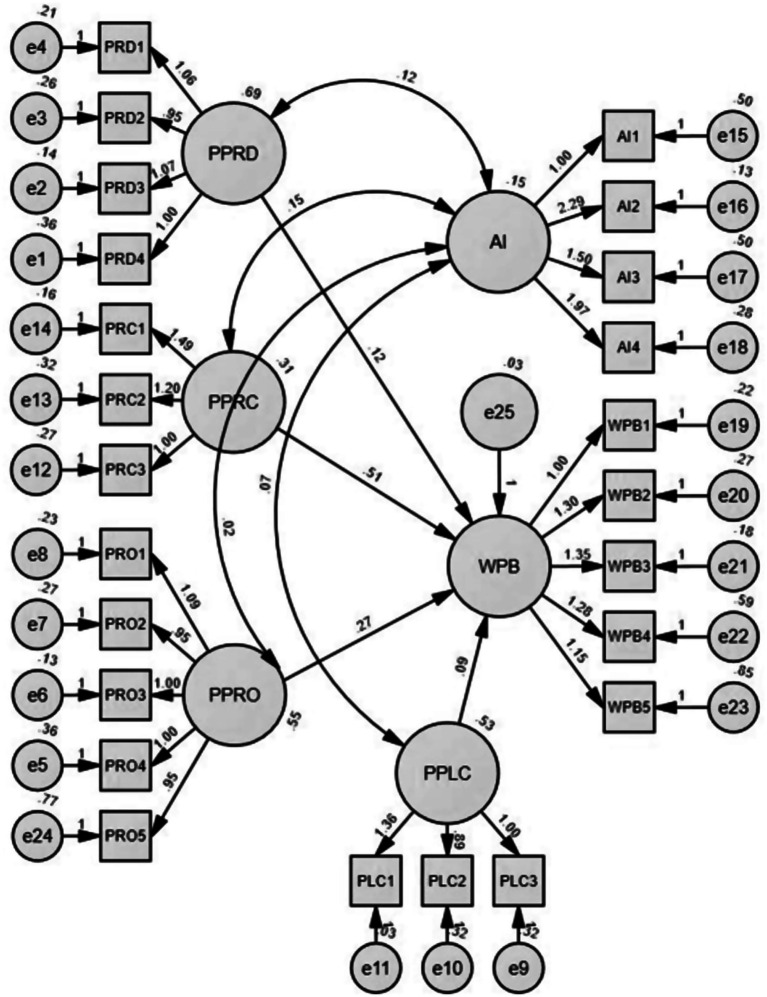
Completed structural equation model.

The path coefficient captured the relationship between PPRO (Pink Promotion) and women’s purchase behavior as 0.27, indicating a positive relationship (Figure 3). These promotional strategies may significantly impact female consumers, as they may relate better to marketing communications tailored explicitly for them, which can turn into purchases. The path coefficient between PPLC and WPB is 0.09, which reflects a positive relationship, but a meager association strength. The authors reason that the relevance of ‘place’ or, in other words, the context of purchase for a woman’s purchasing behavior is less important than other notions considered in the study. As found in the structural model, pink price (0.51) and promotion (0.27) are weightier variables in the model, meaning that they play a significant role in pink marketing strategy. Therefore, it is very important to develop a marketing strategy with both cost and promotion as key components if this strategy targets the women’s market.

### 6.4 AI moderation effect

The addition of AI as a moderating variable in assessing pink constructs and women’s Internet shopping on mobile applications led to noteworthy deviations from the initial R-squared values. For the Pink Product, the AI inclusion guided the R-squared change of 0.699 to 0.714, which means that the model’s explanatory power improved slightly. We observe a more pronounced effect on the pink price, where the AI’s inclusion leads to an R-squared value that jumps significantly from 0.485 to 0.608 (Table 6). This result indicates a clear improvement in explaining the variability in purchasing behavior. Similarly, AI relies mostly on Pink Promotion, as the R-squared value increases from 0.445 to 0.601, which is a substantial increment that clearly shows that AI significantly increases the prediction abilities of promotional techniques. Regarding the Pink Place, a change in the R-squared value from 0.567 to 0.657 through AI is significantly enhanced, indicating that AI filters highly affect where purchases are influenced. This outcome illustrates the vital role of AI in the preview stage of pink marketing elements on mobile consumers’ purchase behavior, which shows its value in comprehending the dynamics of female shoppers’ habits.

**Table 6:** Results of multistep regression analysis.

Independent Variable	Without AI (R Sqrd)	With AI (R Sqrd)	Change (R Sqrd)
Pink Product	0.699	0.714	0.015
Pink Price	0.485	0.608	0.123
Pink Promotion	0.445	0.601	0.156
Pink Place	0.567	0.657	0.090

### 6.5 Model fit indices

The default model fits well because the CMIN/DF ratio was approximately 2.90 (Table 7). The accepted convention is that it should be less than five, but preferably less than three (Kline, 2023). The significant value of the chi-squared test makes the sample size dependent. Still, it represents the character of the model and its usefulness to encompass the complexity of the relationships under study. Moreover, the model is satisfactory in this respect when compared to the independent model, for which the ratio is equal to 6.50. The results can be considered as additional evidence in favor of the model. This further supports the idea that the model adequately fits to provide good estimates for its outcome and may be of value in guiding and building suitable marketing strategies in pink marketing.

**Table 7:** Model fit indices.

Model	NPAR	CMIN	DF	*p*	CMIN/DF
Default model	56	707.672	244	0.000	2.90029508
Saturated model	300	0	0		
Independence model	24	1794.79	276	0.000	6.5028742

### 6.6 Decision on hypotheses

Each hypothesis, H1 through H8, differentiates the pronounced influence of pink marketing factors such as Pink Product (PPRPD), Pink Price (PPRC), Pink Promotion (PPRP), and Pink Place (PPLC) when they jointly vary with AI on the Women’s Mobile Applications Purchase Behavior (WMPB). The *p*-values for each hypothesis are equal to 0.000 (Table 8), which lies below the commonly accepted significance level of 0.05, and consequently supports the established directional relationships. This uniform referral strengthens the comprehensive match between the theoretical model and the observed data. Therefore, it reaffirms the proposed influence of both rose technology elements and the AI middle on the purchasing behavior of women in mobile application settings.

**Table 8:** Summary of decisions on hypotheses.

Hypothesis	Independent		Dependent	*p*-value	Decision
H1	PPRD	→	WPB	0.000	Accepted
H2	PPRC	→	WPB	0.000	Accepted
H3	PPRO	→	WPB	0.000	Accepted
H4	PPLC	→	WPB	0.000	Accepted
H5	PPRDxAI	→	WPB	0.000	Accepted
H6	PPRCxAI	→	WPB	0.000	Accepted
H7	PPROxAI	→	WPB	0.000	Accepted
H8	PPLCxAI	→	WPB	0.000	Accepted

## 7 Discussions, implications, and limitations

The effects of pink products, prices, promotions, and places have been clearly demonstrated. These aspects of the marketing mix have shown how critical they are to mobile application marketers, since they operate in the age of digital consumers. As indicated by Borres et al. (2023), the research findings clearly indicate that besides consumer images, tailored pricing strategies (pink price) also weigh in the purchasing decision of consumers, age matters notwithstanding. However, it is worth noting that aside from the fact that women consumers value product image, cost sensitivity is good enough to influence their buying patterns and decisions. Prices and quality are regularly taken into consideration when it comes to women, since the balance of cost and quality is significant to this demographic (Sohaib et al., 2018). Also pertinent is the influence of a product’s design and attributes on buying preferences, adding to these elements of social psychology and consumer decision-making.

Likewise, the study confirms that the deliberate marketing campaign (pink promotion) is also practical because customers buy advertised products. This is one of the messages that (Evangelista et al., 2022; Andrlić et al., 2023) have been put across, which is that marketing should be aligned with the everyday lives of women. The study also reveals, in support of the pink place theory (Kim and Oh, 2020; Elgarhy and Mohamed, 2023), that the mobile shopping atmosphere has two pronged components: an easy and safe shopping environment, which determines whether women influence mobile shopping choices. The outcomes of this research support the comprehensive literature behind the pink marketing technique, confirming the basis of the marketing mix, which is used as the driving force of women’s purchasing decisions for mobile products and services. The results lay a solid basis for building relevant marketing approaches that would adequately address the female demand for innovative features of mobile commerce.

This study investigates how AI moderates pink marketing and provides a deeper understanding of marketing strategies and consumer behavior. The prominent moderating role of AI throughout the marketing mix from an ancillary to a core element of the 21st-century business market is in accordance with the current marketing literature that discusses the changing nature of technology in consumer markets. In fact, further research on AI-powered pink product strategies could make a similar point as Nawaz et al. (2021), who pointed out that the use of AI for recommendation and personalization of the shopping experience yields benefits to customers. This substantiates the notion that customers’ ability to gather information about merchandise using mobile applications and accumulate the necessary knowledge contributes to women’s buyer behavior.

The power of AI in pink pricing strategies is identical. This study reiterates the findings from Jo (2022) and Bueno and Gallego (2021) the dynamic pricing model of AI, which also benefits from the female market’s unique price sensitivity. AI enables marketers to have data-driven pricing strategies, which customers find to be of great value and a great deal. This, in turn, enhances purchasing behavior. Through the pink-promoting domain, AI’s intermediary influence complements research (Massoudi, 2020) on the significance of customized and enthralled marketing communications. AI-driven promotions through programmed advertising and predictive analysis make promotion content more appealing and relevant, increasing the purchase intent of female customers. How AI affects the place concept of pink marketing is central to the formulation of marketing strategies. According to Al Hamli and Sobaih (2023), the introduction of AI into online stores alters the concept of ‘place,’ as women can thoroughly enjoy shopping in a fully autonomous, secure, and customized environment. This is significant because mobile applications can substantially reshape consumer behavior because of the convenience and context of shopping.

The study outlines that AI not only enriches every element of the pink marketing mix but also synergizes all the elements into a seamless experience which is deeply internalized by female consumers. Harnessing the power of extensive consumer behavior data, AI not only allows the ability to personalize product offerings, set fair dynamic prices, and generate ultra-personalized discounts, but it also enables brands to surface secure and frictionless shopping experiences that satisfy the psychological and functional needs of female consumers. Now, the why of AI impact is the answer to why all the women are looking for personalization, convenience and authenticity in their purchase journeys and traditional marketing fails to deliver. The how then translates into either data-driven or intuitive AI capabilities that tightly couple insights drawing from consumer behavior with real-time adjustments to deliver an uninterrupted, emotionally relevant brand experience across channels. Such a unified approach creates trust and increases purchase intent, making AI-driven pink marketing a game changer in our digital-first world, where decoding consumer’s nuanced needs and responding to them is the key for a low retention rate brand.

The study provides a detailed account of how pink marketing influences women’s mobile app buying behavior, but it has its limitations. A city-focused approach could be a limitation that may overlook rural consumer behavior and technology adoption patterns. Consequently, the diversity of Saudi females in rural areas has not yet been addressed. In particular, the small sample size (mostly middle-aged females) in the study makes it difficult to generalize the findings to other demographics (i.e., either younger or older people who may use mobile applications differently). Moreover, the highly subjective nature of the study could be a cause of response bias. In addition, being cross-sectional, the study failed to account for changes in behaviors over time. The efforts enlighten the gaps, yet areas for future research emerge, including longitudinal studies and broader geographic and demographic sampling, to add to the findings and refine our level of understanding of AI’s role of AI in pink marketing.

## 8 Conclusion and future research

The results confirm that not only do the pink elements of marketing — product, price, promotion, and proper network coverage–influence women’s shopping behavior on mobile applications but the integration of AI also increases it. Such a solid endorsement of all the hypotheses by the respondents suggests a prominent and captivating relationship between the attributes of marketing focusing on women and the specific purchase behaviors of Saudi women in their efforts to technique women-oriented AI technologies. To marketers’ advantage, AI can help not only foretell the female audience’s needs and preferences but also make them engage in personalized experiences. However, this research fills a crucial gap in the publication’s realm, which is focused on emerging markets, and presents a compelling case for the inclusion of AI in pink marketing strategies. Challenges include the focus on urban settings and the fact that some of the data entered through self-reporting are present, but they allow for a solid first step that can be enhanced in further research. The findings have great significance for both marketers and app developers who address Saudi women, as they plan their marketing activities in a way that will be gender-focused as well as data-driven and tech-savvy.

Future research should expand this pink marketing study by exploring the differences caused by different cultural settings and age groups. A comparative analysis of urban and rural purchasing habits should be conducted to acquire a precise global picture. Such longitudinal studies should present time concerns, taking into account the increasing number of new technologies. To be accurate, we need to conduct quantitative methodologies where we can obtain data, but at the same time, we should employ qualitative methods such as interviews or focus groups to supplement the quantitative data that we obtain. The next step is hypothesis testing, in which the effectiveness of particular AI features, such as chatbots and augmented reality, are analyzed to determine how they enhance women’s shopping experience. In addition, developing an analysis of male opinions about pink marketing may allow a target market to draw up a more comprehensive impact of its influence on the general market. Along with machine learning algorithms for predictive analytics and the ethical dilemmas arising from AI in consumer data accumulation, there is also a broad topic that makes the exploration quite interesting. By exploiting various techniques, future studies can construct a more articulate and operational solution for improving pink marketing strategies in this contemporary digital age.

## Data Availability

The raw data supporting the conclusions of this article will be made available by the authors, without undue reservation.
