# Supplementary material for: The effect of AI on pink marketing: the case of women’s purchasing behavior using mobile applications
Source: Front Artif Intell. 2024 Nov 18;7:1502580. doi: 10.3389/frai.2024.1502580 (PMC11609155; doi:10.3389/frai.2024.1502580)
Supplement: Supplementary file 1 [file Data_Sheet_1.docx]

# **Appendix A.**

# **Research Questionnaire**

| **Pink Product** | **Source** |
| --- | --- |
| 1. I am attracted to mobile apps that provide products with designs targeting women in particular. | [11] |
| 2. The singularity of a product designed for women determines whether I use a mobile app to obtain it. | [12] |
| 3. As a woman, I feel more content with my acquisition when the product suits my personal preference. | [14] |
| 4. I’m loyal to the mobile applications which provide products with features that are friendly for women. | [12] |
| **Pink Price** | **Source** |
| 1. I tend to make purchases from an application that provides reasonable prices on ladies’ products. | [15] |
| 2. I am affected by discounts and special offers on women’s products in mobile apps when purchasing. | [16] |
| 3. The clarity of pricing in mobile apps makes me confident and choose them for buying. | [16] |
| **Pink Promotion** | **Source** |
| 1. Targeted mobile app promotions for women goods manage to capture my eyes and have impact upon my consumerism. | [9] |
| 2. I like mobile apps that offer women product oriented promotional content. | [11] |
| 3. I am more convinced by mobile app ads that correspond with women’s values and lifestyles. | [15] |
| **Pink Place** | **Source** |
| 1. I like buying through apps that offer female-friendly shopping environment. | [14] |
| 2. The ease of finding women-specific products on a mobile app is a key factor for me in choosing to use it. | [14] |
| 3. I am more likely to shop at a mobile application that provides an effortless and safe checkout process for women’s products. | [14] |
| 4. I treasure mobile applications that provide the comfortable women products return and exchange cycles. | [14] |
| 5. I like mobile apps that offer comprehensive product information aimed at women. | [18] |
| **Artificial Intelligence** | **Source** |
| 1. AI personalized product recommendations on mobile applications improve my shopping experience. | [20] |
| 2. The AI-generated elements that enable me to see products increase my tendency of buying something at a mobile app. | [20] |
| 3. The consumer can easily shop for women’s products using the chatbots and virtual assistants on mobile applications. | [20] |
| 4. I rely on AI-powered mobile apps to provide women-oriented offers and promotions. | [18] |
| **Women Mobile Applications Purchase Behavior** | **Source** |
| 1. I am a regular buyer via the apps that are targeted towards women. | [21] |
| 2. The quality of the mobile application in catering to my needs as a woman dictates my decision to use it a shopping source. | [22] |
| 3. I usually invest more in mobile apps that offer individualized shopping options for women. | [21] |
| 4. I am more likely to recommend a mobile app if it manages to satisfy women specific shopping preferences. | [23] |
| 5. I am dedicated to mobile apps that continuously enhance their shopping experience for the benefit of women through customer evaluations. | [22] |
